# WNT7A Regulation by miR-15b in Ovarian Cancer

**DOI:** 10.1371/journal.pone.0156109

**Published:** 2016-05-19

**Authors:** James A. MacLean, Mandy L. King, Hiroshi Okuda, Kanako Hayashi

**Affiliations:** 1 Department of Physiology, Southern Illinois University School of Medicine, Carbondale, Illinois, United States of America; 2 Laboratory for Malignancy Control Research, Kyoto University Graduate School of Medicine, Kyoto, Japan; The University of Texas MD Anderson Cancer Center, UNITED STATES

## Abstract

WNT signaling is well known to play an important role in the regulation of development, cell proliferation and cell differentiation in a wide variety of normal and cancerous tissues. Despite the wealth of knowledge concerning when and where various *WNT* genes are expressed and downstream events under their control, there is surprisingly little published evidence of how they are regulated. We have recently reported that aberrant WNT7A is observed in serous ovarian carcinomas, and WNT7A is the sole ligand accelerating ovarian tumor progression through CTNNB1 (β-catenin)/TCF signaling in the absence of CTNNB1 mutations. In the present study, we report that *WNT7A* is a direct target of *miR-15b* in ovarian cancer. We showed that a luciferase reporter containing the putative binding site of *miR-15b* in the *WNT7A* 3’-UTR was significantly repressed by *miR-15b*. Mutation of the putative binding site of *miR-15b* in the *WNT7A* 3’-UTR restored luciferase activity. Furthermore, *miR-15b* was able to repress increased levels of TOPFLASH activity by WNT7A, but not those induced by S33Y. Additionally, *miR-15b* dose-dependently decreased *WNT7A* expression. When we evaluated the prognostic impact of *WNT7A* and *miR-15b* expression using TCGA datasets, a significant inverse correlation in which high-expression of *WNT7A* and low-expression of *miR-15b* was associated with reduced survival rates of ovarian cancer patients. Treatment with decitabine dose-dependently increased *miR-15b* expression, and silencing of *DNMT1* significantly increased *miR-15b* expression. These results suggest that *WNT7A* is post-transcriptionally regulated by *miR-15b*, which could be down-regulated by promoter hypermethylation, potentially via DNMT1, in ovarian cancer.

## Introduction

MicroRNA (miRNAs) are small noncoding RNAs that regulate gene expression by post-transcriptional mRNA silencing. The processes regulated by miRNAs involve a variety of biological pathways, and their dysregulation is a common feature of human cancer [1–3]. The miR-15 family includes six highly conserved members, *miR-15a*, *miR-15b*, *miR-16-1*, *miR-16-2*, *miR-195* and *miR-497*, which are clustered on three different chromosomes [4, 5]. The *miR-15a/16-1* cluster, was originally reported as the target of 13q14 deletions or downregulation in chronic Lymphocytic leukemia (CLL) [6]. Specifically, *miR-15a* and *miR-16-1* directly regulate *BCL2*, which is an anti-apoptotic oncogene [7], and hence act as tumor suppressors by inducing apoptosis [8]. Further studies have shown that *miR-15a* and *miR-16-1* act as putative tumor suppressors by targeting *BCL2*, *BMI1 CCND1*, *MCL1* and *WNT3A* in CLL, melanoma, as well as colon, bladder, ovarian and prostate cancers [4, 9, 10]. The *miR-15b*/*miR-16-2* cluster, which is located in 3q25, has also been reported to act in tumor suppression by targeting *BCL2*, *BM1*, *CCND1* and *SUZ12* [11–13]. Reduction of *miR-15b* was observed in CLL, melanoma, gastric and chemoresistant tongue cancer, as well as cancer stem cells [11–15]. Deletion of *miR-15b* and *miR-16-2* promotes B-cell pathogenesis [11]. Thus, the direct targets of the miR-15 family members are likely to be critical oncogenes.

We have recently reported that the upregulation of WNT7A (uniquely among 19 WNT ligands) results in accelerated development and progression of ovarian cancer (OvCa), and plays a critical role in tumor progression mediated by the WNT7A/CTNNB1 signaling pathway [16, 17]. Our studies further indicate that *FGF1* is a direct downstream target of WNT7A/CTNNB1 signaling, and that this pathway has potential as a therapeutic OvCa target [16]. One of our findings clearly showed that high expression of *WNT7A* and *FGF1* were correlated in OvCa, especially in serous carcinomas, and poor overall patient survival [16]. Thus, WNT7A encodes a potent oncogenic factor of relevance to OvCa. However, we still do not know the answer as to why WNT7A becomes specifically overexpressed in serous OvCa.

In the present study, we report that abundant WNT7A, present in OvCa, is post-transcriptionally down-regulated by *miR-15b*. In support of its role in cancer inhibition, OvCa patients had poor overall survival rate in the group with high expression of *WNT7A* and low expression of *miR-15b*. Furthermore, our results indicate that DNMT1 modulates *miR-15b* transcription through promoter methylation.

## Materials and Methods

### Reagents and plasmids

The 3’-UTR segments of the endogenous *WNT7A* gene and its mutant form were amplified by PCR, and subcloned into the pMIR-REPORT vector (Thermo Fisher) using the SpeI and HindIII restriction sites to generate pMIR-*WNT7A* and pMIR-*WNT7A* mutation 3’-UTR-containing plasmids. Decitabine (5’aza-2’deoxycytidine, aka: 5-aza-2’-dC), *mir*Vana miRNA mimic (negative control and hsa-miR-15b-5p), DNMT1 siRNAs, pre-miR-15b expression constructs and Dual Luciferase Reporter Assay System were purchased from Cayman Chemical, Thermo Fisher, Qiagen, System Biosciences and Promega, respectively.

### Cell lines

OVCAR3, OVCAR5, SKOV3, and ES2 cells were purchased from the American Type Culture Collection (ATCC, Manassas, VA, USA). OVCAR4 cells were gifted from Dr. Joanna Burdette (University of Illinois at Chicago). KURAMOCHI, OVKATE and OVSAHO were purchased from the JCRB cell bank (Osaka, Japan). SKOV3.ip1 cells were purchased from the cell bank at The University of Texas MD Anderson Cancer Center. All cells were authenticated by short tandem repeat (STR) analysis and passaged within 6 months of receipt. All cells were tested routinely for cell proliferation and BrdU incorporation as well as mycoplasma contamination. All cell lines exhibited similar morphology, characteristic growth rates, and remained negative for mycoplasma contamination throughout all experiments. OVCAR4, KURAMOCHI, OVKATE and OVSAHO cells were grown in RPMI 1640 with 10% FBS and penicillin/streptomycin, and other cells were cultured in DMEM with 10% FBS, 2mM glutamine and penicillin/streptomycin. All cell lines were grown in a humidified incubator at 37°C and constant 5% CO_2_.

### Quantitative real-time PCR (qPCR) assay

Total RNA was extracted from cells, and cDNA was synthesized from total RNA using the High-Capacity cDNA Reverse Transcription Kit from Thermo Fisher. Relative gene expression was determined by SYBR green (Bio Rad) incorporation using a Bio-Rad myCycler as described previously [18]. Micro RNA was extracted from cells using Pure Link miRNA isolation kit (Thermo Fisher), and cDNA was synthesized by using miScript II RT Kit (Qiagen). Relative miRNA expression was determined by miScript SYBR Green PCR kit (Qiagen), and miR-15b and SNORD68 specific primers (Qiagen). A table of oligonucleotides used for each gene is presented in S1 Table.

### Cell proliferation and adhesion assays

Cell proliferation, adhesion and migration assays were performed following our previously described methods [16, 17]. To assess cell proliferation, cells were seeded in 24-well plates, and counted 24, 48 and 72 hours by Countess II FL Automated Cell Counter (Thermo Fisher) with trypan blue exclusion. To assess cell adhesion, cells were seeded in 24-well plates and harvested after 4 h incubation.

### Statistical analyses

All experimental data were subjected to one-way ANOVA and differences between individual means were tested by a Tukey multiple-range test using Prism 5.0 (Graphpad). QPCR data were corrected for differences in sample loading using the *RPL19* data as a covariate. Tests of significance were performed using the appropriate error terms according to the expectation of the mean squares for error. A p-value of 0.05 or less was considered significant. Data are presented as means with standard error of the means (SEM). The Kaplan-Meier method was used to calculate the survival rates and was evaluated by the log-rank test using a TCGA dataset, TCGA-OV that contained 554 primary ovarian tumors with completed data sets, was selected for survival analysis.

## Results

### WNT7A expression in OvCa cells depending on genetic background or features

When we reported the clinical significance of WNT7A during malignant transformation of OvCa, our results showed that *WNT7A* was highly expressed in serous carcinomas, the most common/aggressive subtype of OvCa [16, 17]. Thus, aberrant WNT7A could be induced by abnormal genetic background or correlated with aggressive characters in OvCa. When we examined *WNT7A* expression levels in OvCa cells, abundant *WNT7A* (>1000 fold) was observed in invasive or high-grade serous OvCa cells (SKOV3.ip1, KURAMOCHI, OVCAR4 and OVSAHO, S1 Fig). Note: KURAMOCHI, OVCAR4 and OVSAHO cells have been recently confirmed as high-grade serous OvCa cell lines by genomic profiling [19]. SKOV3.ip1 cells possess highly invasive and metastatic features as these cells are isolated from ascites fluids [20]. ES2, OVCAR3 and OVCAR5 cells possess genomic profiles that are partially similar to serous OvCa tumors.

### WNT7A is a direct target of miR-15b

**Fig 1:**
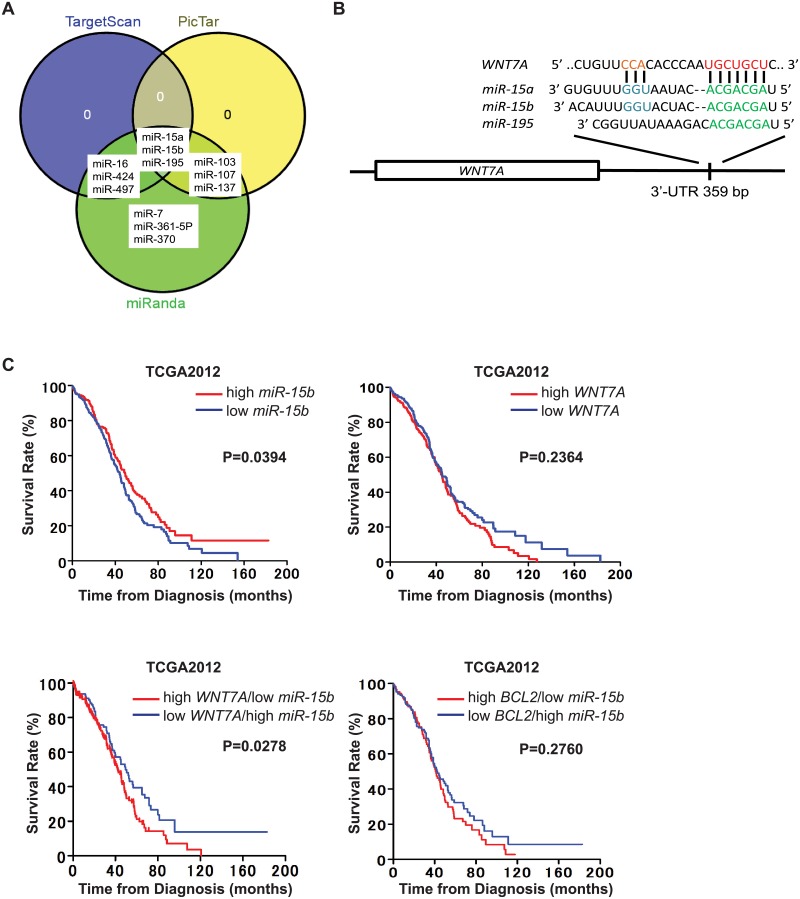
(A) Bioinformatics prediction of miRNA interaction with seeded sequences from the 3’-UTR of *WNT7A* using three different algorithms. (B) Schematic of the putative *miR-15b* binding sequence in the *WNT7A* 3’-UTR. (C) *WNT7A* or *BCL2* and *miR-15b* expression inversely correlates with survival calculated with TCGA-OV datasets (total n = 554, high *WNT7A*/*miR-15b*, n = 132; high *WNT7A*/low *miR-15b*, n = 147; low *WNT7A*/high *miR-15b*, n = 146; low *WNT7A*/*miR-15b*, n = 129) by Kaplan-Meier method using Prism 5.0. The P-value was determined by the long rank test.

We examined transcriptional activation of the *WNT7A* promoter by using luciferase reporter assays, however, our deletion analyses did not reveal any critical sites or potential regulators within a distance of 10 kb up- or down-stream of the *WNT7A* promoter (data not shown). Therefore, we subjected the *WNT7A* 3’-UTR to an in silico analysis using 3 different algorithms (TargetScan, PicTar and miRanda) to identify putative miRNA seed-matching sequences. All three search engines detected *miR-15a*, *miR-15b* and *miR-195* consensus binding sequences (Fig 1A) in the 3’-UTR of *WNT7A* (Fig 1B). We next evaluated the prognostic impact of *WNT7A* and/or *miR-15b* expression using TCGA datasets (total patient number is 554). While high-expression of *miR-15b* (n = 278) showed a good prognosis (P = 0.0394) compared with low-expression of *miR-15b* (n = 276), *WNT7A* did not show any correlation (high-expression of WNT7A, n = 279 vs low = expression of WNT7A, n = 275, P = 0.2364). A significant inverse correlation in which high-expression of *WNT7A* and low-expression of *miR-15b* (n = 147 vs n = 146 of low-expression of *WNT7A* and high-expression of *miR-15b*) was associated with reduced survival rate of ovarian cancer patients by log-rank test (P = 0.0297, Fig 1C). NOTE: high-expression of *WNT7A*/*miR-15b* n = 132 and low-expression of *WNT7A*/*miR-15b* n = 129 were not included in inverse correlation analysis. However, no inverse correlation was seen between *WNT7A* and *miR-15a* or *miR-197* (data not shown). Therefore, we focused on *miR-15b* for further analyses. Additionally, we examined the inverse correlation between *BCL2* and *miR-15b*, as *BCL2* is one of the well-known targets of miR-15 family and acts as an oncogene. However, no significant association between *BCL2* and *miR-15b* concerning the survival rate of ovarian cancer patients using TCGA-OV datasets (P = 0.2760) was observed, suggesting that *WNT7A* is a more relevant critical target of *miR-15b* with respect to OvCa.

**Fig 2:**
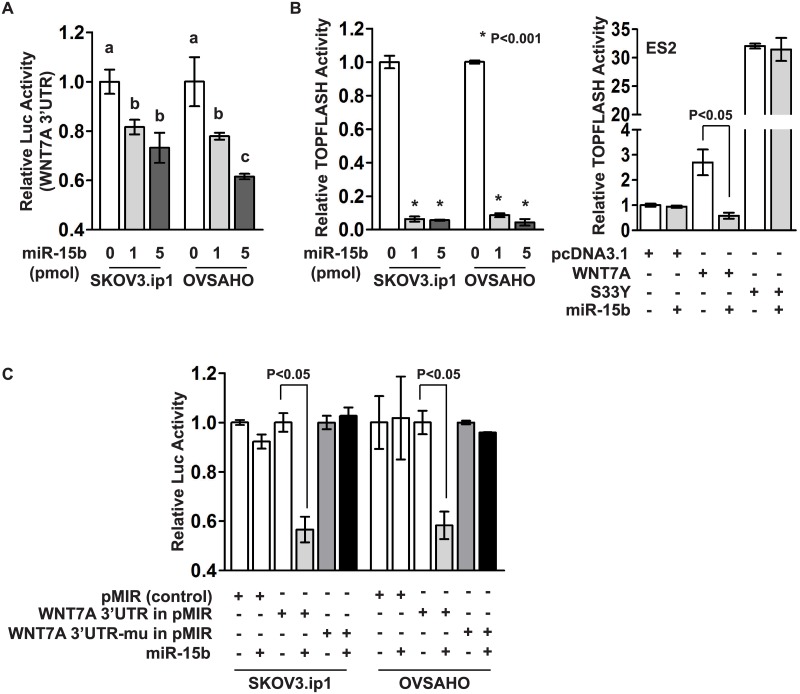
(A) Luciferase reporter analysis of the *WNT7A* 3’-UTR in SKOV3.ip1 and OVSAHO cells 48 hours after transfection of *miR-15b* or negative control mimic. Different letters denote reporter activities that have statistically significant (P<0.05) differences in mean activities. (B) TOPFLASH reporter analysis in SKOV3.ip1 and OVSAHO cells 48 hours after transfection of *miR-15b* or negative control mimic (left). TOPFLASH reporter analysis in ES2 cells 48 hours after transfection of *miR-15b* mimic, WNT7A or S33Y with negative controls (mimic or pcDNA3.1, right). (C) Luciferase reporter analysis of the *WNT7A* 3’-UTR, mutation of *WNT7A* 3’-UTR or pMIR-REPORT vector in SKOV3.ip1 and OVSAHO cells 48 hours after transfection of *miR-15b* or negative control mimic.

**Fig 3:**
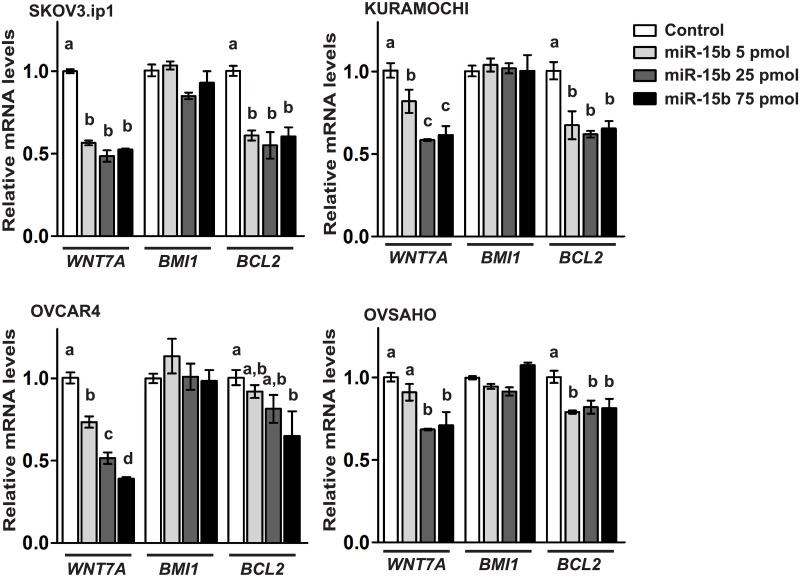
Relative *WNT7A*, *BMI1* and *BCL2* expression was assessed by qPCR in OvCa cells after transfection of *miR-15b* or control mimic. Data were set to background level of 1 with respect to control levels. Different letters denote transcripts that have statistically significant (P<0.05) differences in mean expression levels.

We used TargetScan 7.0 [21], to identify *miR-15b* target sites within the *WNT7A* 3’-UTR and found one putative *miR-15b* binding site (Fig 1B). To examine whether *miR-15b* binds to this sequence, OvCa cells were transfected with pMIR-REPORT plasmid containing the putative binding site of *miR-15b* in the *WNT7A* 3’-UTR, and *mir*Vana miRNA mimic (negative control or miR-15b), and then luciferase reporter activity was measured (Fig 2A). Luciferase activity was significantly repressed by *miR-15b* compared with negative control. In the previous study, we have shown that WNT7A activates the canonical CTNNB1 signaling pathway [16, 17]. In order to determine whether *miR-15b* inhibits WNT7A’s action, we examined CTNNB1 mediated transcriptional activity with TOPFLASH reporter construct (Fig 2B). We found that *miR-15b* significantly repressed TOPFLASH reporter activity. When TOPFLASH activity was increased by WNT7A or S33Y-mutated CTNNB1 (an established positive control for activation of the TOPFLASH reporter) in ES2 cells, that lowly or undetectably possess endogenous WNT7A, *miR-15b* was able to repress increased levels of TOPFLASH activity by WNT7A, but not those induced by S33Y (Fig 2B). Furthermore, mutation of the putative binding site of *miR-15b* in the *WNT7A* 3’-UTR (5’TGCTGCT3’ to 5’TaaTGCT3’) restored the luciferase activity previously repressed by *miR-15b* (Fig 2C). In support of these findings, *miR-15b* dose-dependently decreased *WNT7A* mRNA levels in OvCa cells (Fig 3). These results suggest that *WNT7A* expression is directly regulated by *miR-15b* in OvCa. Because *BMI1* and *BCL2* have been reported as target genes of *miR-15b* in cancer [12, 13], their mRNA levels in OvCa were also examined. While *miR-15b* was able to decrease *BCL2*, *BMI1* was not regulated by *miR-15b* in OvCa cells.

### MiR-15b inhibits cell proliferation and adhesion

**Fig 4:**
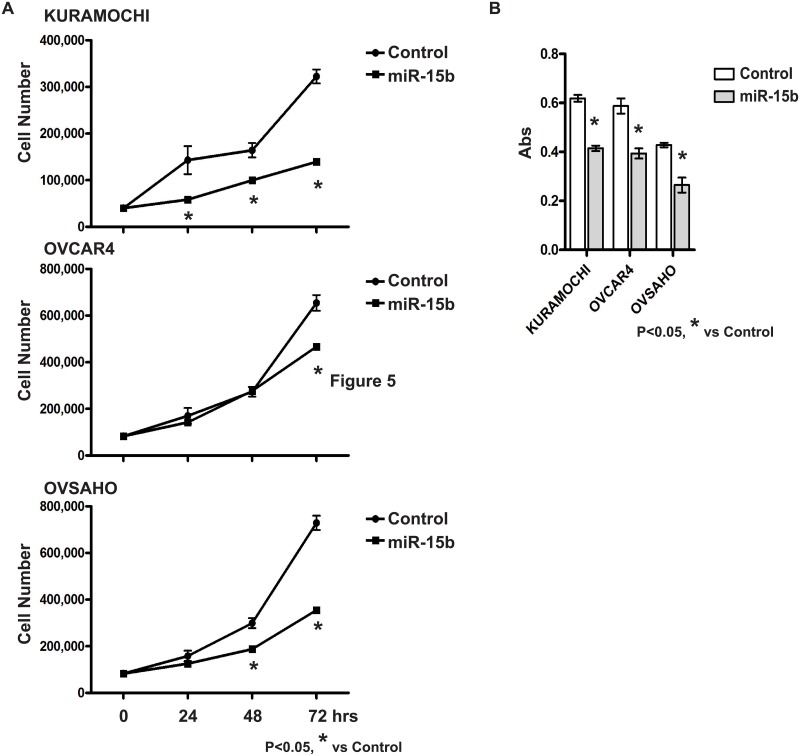
(A) Cell proliferation or (B) adhesion with either *miR-15b* expressing or control cells.

The role of *miR-15b* as a tumor suppressor has been characterized, as loss of *miR-15b* in mice leads to the development of B-cell malignancy [11], and overexpression of *miR-15b* suppresses metastasis dissemination using tongue cancer xenografts [12]. In the present study, *miR-15b* overexpressing OvCa cells were time-dependently less proliferative (Fig 4A), and reduced cell adhesion (Fig 4B), indicating that *miR-15b* also acts as tumor suppressor in OvCa.

### MiR-15b is regulated by promoter hypermethylation

**Fig 5:**
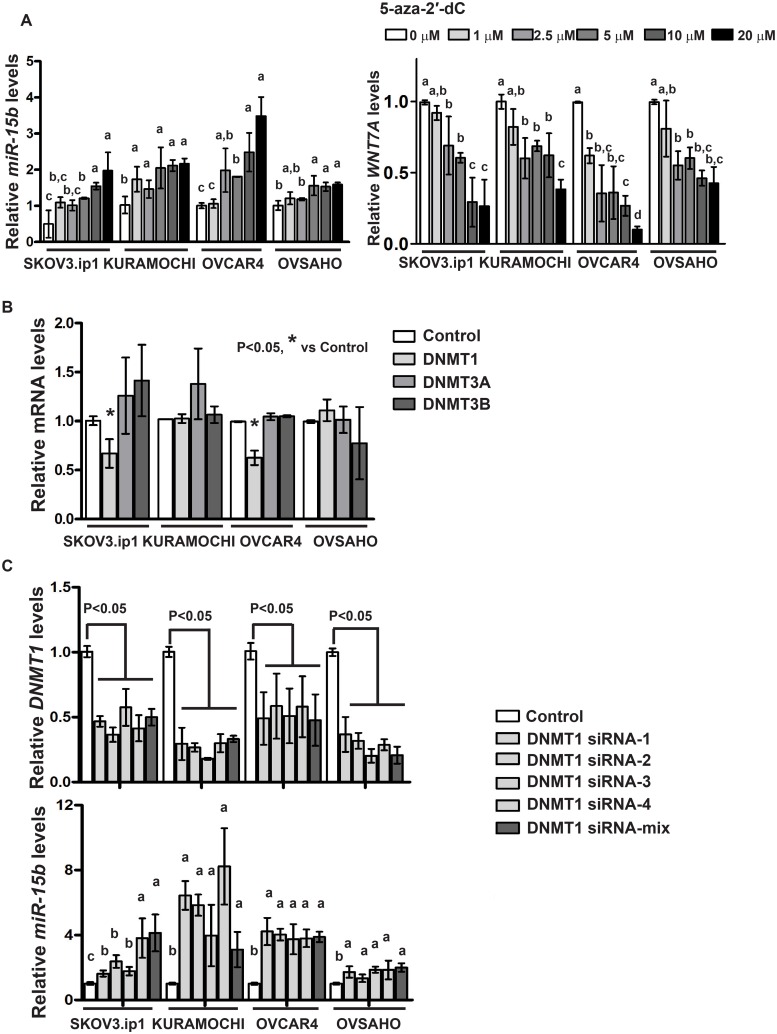
(A) 5-aza-2’-dC treatment induces *miR-15b* expression (left) and decreases *WNT7A* expression (right) in OvCa cells. The cells were treated with 5-aza-2’-dC for 3 days, and *miR-15b* or *WNT7A* was assessed by qPCR compared to 0 μM control (set to background level of 1 in each cell). Different letters denote transcripts that have statistically significant (P<0.05) differences in mean expression levels. (B) *DNMT1*, *DNMT3A* and *DNMT3B* expression levels were assessed in OvCa cells after 5-aza-2’-dC (5 μM) treatment for 3 days. (C) The expression levels of *DNMT1* (upper) and *miR-15b* (bottom) were assessed in OvCa cells transfected with DNMT1 siRNA 1–4, DNMT1 siRNA mix or scramble control. Different letters denote transcripts that have statistically significant (P<0.05) differences in mean expression levels.

Our present results suggest that abundant expression of *WNT7A* is likely induced due to downregulation of *miR-15b*. The prognostic impact of OvCa patients with inverse correlation of *WNT7A* and *miR-15b* further support this hypothesis. Therefore, we tested the possibility that *miR-15b* might be down-regulated in OvCa through promoter hypermethylation. Treatment with an inhibitor of DNMTs, 5-aza-2’dC, dose-dependently increased *miR-15b* expression and decreased *WNT7A* expression in OvCa cells (Fig 5A). When we examined the expression levels of three active DNMT isoforms (*DNMT1*, *DNMT3A* and *DNMT3B*) in 5-aza-2’dC (5 μM) treated OvCa cells, only *DNMT1* was reduced in SKOV3.ip1 and OVCAR4 cells (Fig 5B), indicating that DNMT1 could be functional to methylate *miR-15b*. Indeed, silencing of *DNMT1* significantly increased *miR-15b* expression in OvCa cells (Fig 5C), suggesting that *miR-15b* is potentially down-regulated, especially in high WNT7A-expressing cells, by promoter hypermethylation via DNMT1 in OvCa.

## Discussion

WNT signaling is well known to play an important role in cancer biology [22]. While CTNNB1 is the key mediator of WNT signaling, we have demonstrated that WNT7A is the sole ligand activating intact CTNNB1/TCF signaling (i.e. within cells lacking activation by mutation of CTNNB1), especially the serous OvCa subtype [16, 17]. Despite the wealth of knowledge concerning the expression of various WNT ligands and downstream events under their control, there is surprisingly little published evidence of how *WNT* genes are regulated, and why some members are upregulated in specific tumor types. Recently, *WNT3A*, which promotes tumorigenesis via accelerated cellular proliferation and invasion [23], is found to be directly regulated by the *miR-15a/16-1* cluster, and upregulation of *WNT3A* is inversely correlated with decreased *miR-15a* and *miR-16-1* in advanced prostate tumors [10]. In the present study, bioinformatics seed-matching programs identified three highly conserved miR-15 family members, *miR-15a*, *miR-15b* and *miR-195*, which could be critical regulators of *WNT7A*. However, neither *miR-15a* nor *miR-195* exhibited significant inverse correlations with *WNT7A* in the OvCa patient survival data sets. Thus, *WNT7A* expression is most likely subject to regulation by *miR-15b* in OvCa.

Previous work in CLL has demonstrated the tumor suppressor activity of the *miR-15a/16-1* cluster is dependent on repression of *BCL2* [8]. *BCL2* has also been reported as a direct target of *miR-15b* using a model of drug resistant gastric cancer cells [13]. It has been characterized that *miR-15b/16-2* knockout mice develop B-cell malignancy, whereas BCL2 faintly up-regulated in B-cells from knockout mice [11]. Our results showed that *miR-15b* repressed *BCL2* expression in OvCa cells. However, no inverse correlation between *BCL2* and *miR-15b* was observed in the analysis of patient survival in OvCa. These results suggest that regulation of BCL2 by *miR-15b* has less impact on ovarian tumorigenesis.

The actions of *miR-15b* as a tumor suppressor have been clearly demonstrated in the pathogenesis of B-cells in CLL [11]. In addition, overexpression of *miR-15b* inhibits metastasis dissemination via epithelial-mesenchymal transition in the model of chemoresistant tongue cancer cell xenografts [12]. Downregulation of *miR-15b* occurs in breast cancer stem cells, and overexpression of *miR-15b* inhibits their growth and differentiation [15]. Inhibition of cell proliferation targeting of cyclin D1, and induction of apoptosis by *miR-15b* have also been reported [11–14, 24]. Our results further support *miR-15b*’s tumor suppressor function, and add inhibition of OvCa cell proliferation and cell adhesion to its list of relevant tumors.

In the present study, we showed that 5-aza-2’-dC, an inhibitor of DNMT activity, increased *miR-15b* expression. Similarly, decreased *DNMT1* was observed by the treatment of 5-aza-2’-dC, and silencing of *DNMT1* increased *miR-15b* in OvCa cells. Increased DNMT1 levels have been reported in OvCa, with lower expression in primary stage I/II tumors and peak expression occurring at stage III/IV [25]. DNMT1-mediated promoter hypermethylation induces reduction of E-cadherin and progresses invasive feature of OvCa [26]. There is one report examining the correlation between alteration in copy number at the chromosomal location of *miR-15b* and the changes in *miR-15b* promoter methylation status for breast, ovarian, head and neck, lung and kidney cancer using TCGA data sets [27]. This group found significant correlation between *miR-15b* expression and copy number variation at those loci, as well as between *miR-15b* expression and methylation levels in the relevant cancer types and the pooled data from all cancer types. Note: No methylation data are available for ovarian cancer in TCGA (only expression and copy number alteration). Furthermore, the promoter region of *miR-16-2*, which is present in a cluster with *miR-15b* located on chromosome 3q25, is methylated in polycythemia vera CD34+ cells [28]. Although the epigenetic regulation of *miR-15b* in OvCa remains to be investigated, DNMT1 may be one of the regulators to suppress *miR-15b*.

In summary, we found that *WNT7A* is directly regulated by *miR-15b* in OvCa. As WNT7A activates tumor growth and progression in OvCa via the WNT7A/CTNNB1 signaling pathway [16, 17], downregulation of *miR-15b* allows aberrant *WNT7A* expression is further support the impact of WNT7A and its mechanisms in OvCa.

## Supporting Information

S1 Fig Relative *WNT7A* expression was assessed by qPCR in OvCa cells. Data are expressed as fold above ES2 expression levels, which were the near background and arbitrarily set to 1.
(EPS)

S1 Table Primers for qPCR (PDF)
