# Supplementary material for: WNT7A Regulation by miR-15b in Ovarian Cancer
Source: PLoS One. 2016 May 19;11(5):e0156109. doi: 10.1371/journal.pone.0156109 (PMC4873135; doi:10.1371/journal.pone.0156109)
Supplement: S1 Table — (PDF) [file pone.0156109.s002.pdf]

Supplemental Table S1. Primers for qPCR.

Primer		Sequence of forward and reverse primers 5'-3'
WNT7A	For	CTGGAACTGCTCTGCACTGGGA
	Rev	GTACAGGCAGCTGTGATGGCGT
BMI1	For	ACTTCATTGATGCCACAACC
	Rev	CAGAAGGATGAGCTGCATAA
BCL2	For	GCAATTCCGCATTTAATTCATGG
	Rev	GAAACAGGCCACGTAAAGCAAC
DNMT1	For	CCTAGCCCCAGGATTACAAGG
	Rev	ACTCATCCGATTTGGCTCTTTC
DNMT3A	For	CCGATGCTGGGGACAAGAAT
	Rev	CCCGTCATCCACCAAGACAC
DNMT3B	For	AGGGAAGACTCGATCCTCGTC
	Rev	GTGTGTAGCTTAGCAGACTGG
RPL19	For	AGCCTGTGACGGTCCATTCC
	Rev	CGGCGCAAAATCCTCATTCT
